# Differences in long-term memory stability and AmCREB level between forward and backward conditioned honeybees *(Apis mellifera)*

**DOI:** 10.3389/fnbeh.2015.00091

**Published:** 2015-04-27

**Authors:** Johannes Felsenberg, Yan Dyck, Janina Feige, Jenny Ludwig, Jenny Aino Plath, Anja Froese, Melanie Karrenbrock, Anna Nölle, Karin Heufelder, Dorothea Eisenhardt

**Affiliations:** FB Biologie, Pharmazie, Chemie, Institut für Biologie, Neurobiologie, Freie Universität BerlinBerlin, Germany

**Keywords:** classical conditioning, backward conditioning, long-term memory, transcription, CREB, proteasome, ubiquitin

## Abstract

In classical conditioning a predictive relationship between a neutral stimulus (conditioned stimulus; CS) and a meaningful stimulus (unconditioned stimulus; US) is learned when the CS precedes the US. In backward conditioning the sequence of the stimuli is reversed. In this situation animals might learn that the CS signals the end or the absence of the US. In honeybees 30 min and 24 h following backward conditioning a memory for the excitatory and inhibitory properties of the CS could be retrieved, but it remains unclear whether a late long-term memory is formed that can be retrieved 72 h following backward conditioning. Here we examine this question by studying late long-term memory formation in forward and backward conditioning of the proboscis extension response (PER). We report a difference in the stability of memory formed upon forward and backward conditioning with the same number of conditioning trials. We demonstrate a transcription-dependent memory 72 h after forward conditioning but do not observe a 72 h memory after backward conditioning. Moreover we find that protein degradation is differentially involved in memory formation following these two conditioning protocols. We report differences in the level of a transcription factor, the cAMP response element binding protein (CREB) known to induce transcription underlying long-term memory formation, following forward and backward conditioning. Our results suggest that these alterations in CREB levels might be regulated by the proteasome. We propose that the differences observed are due to the sequence of stimulus presentation between forward and backward conditioning and not to differences in the strength of the association of both stimuli.

## Introduction

Learning about the predictive relationship between a neutral stimulus and a meaningful event is crucial for animals and allows them to beneficially adjust their future behavior to environmental changes. In the framework of classical conditioning a predictive relationship between a neutral stimulus (conditioned stimulus; CS) and a meaningful stimulus (unconditioned stimulus; US) is learned when the CS precedes the US. During such a forward conditioning procedure the CS acquires the capacity to elicit a behavioral response, the conditioned response (CR), which is often similar to the unconditioned response (UR) evoked by the US alone. Thus the CS acquires excitatory properties during forward conditioning (Pavlov, 1927). In backward conditioning, in contrast, the sequence of the stimuli is reversed. In this situation animals might learn that the CS signals the end or the absence of the US. Accordingly, the CS does not elicit behavior but rather inhibits behavior towards the US. In this case the CS acquires inhibitory properties (Pavlov, 1927; Moscovitch and LoLordo, 1968).

Previous studies on backward conditioning used a retardation-of-acquisition assay to demonstrate the CS’ inhibitory properties (Hammond, 1968; Rescorla, 1969; Papini and Bitterman, 1993). In this assay forward conditioning follows backward conditioning. If the CS acquired inhibitory properties during backward conditioning, the CS response during subsequent forward conditioning is retarded. On the contrary, if excitatory properties are acquired the response during forward conditioning should be enhanced.

Following backward conditioning two memories can be formed: one memory about the CS’ inhibitory properties and one memory about its excitatory properties (Domjan and Siegel, 1971; Keith-Lucas and Guttman, 1975; Heth, 1976; Williams et al., 1992; Cole and Miller, 1999; Urushihara, 2004; Felsenberg et al., 2013). The formation of the two opposing memories following backward conditioning has been demonstrated not only in vertebrates, but also in the honeybee *(Apis mellifera)*, an established insect model organism for the study of learning and memory formation (Eisenhardt, 2014). Work on honeybees demonstrates that a memory for the excitatory and inhibitory properties of the CS can be retrieved 30 min and 24 h after backward conditioning (Felsenberg et al., 2013). While memory formation following forward conditioning is well characterized in honeybees, both behaviorally and at the level of the underlying molecular mechanisms (reviewed in Eisenhardt, 2006, 2014), the stability and molecular mechanisms underlying memory formation upon backward conditioning remain unknown. So, it is unclear whether memories formed after forward and backward conditioning are equally long lasting and whether the same molecular mechanisms are involved. Here we examine the stability of long-term memories following forward and backward conditioning focusing on transcription-dependent late long-term memories (lLTM) that can be retrieved 72 h after conditioning and study the underlying molecular mechanisms.

## Materials and Methods

Honeybees were handled as described in Felsenberg et al. (2011). In detail, forager bees were caught in the afternoon at about 2:00 p.m. in front of the beehives kept at the Freie Universität Berlin. Each bee was harnessed in a plastic tube and subsequently fed between 4:00 and 5:00 p.m. to satiation with 0.88 M sucrose solution. Bees were maintained overnight in a dark, humid box. Experiments started at the next morning (9:30 a.m.) by moving the harnessed bees from the box next to the experimental set-up. Thirty minutes later bees were injected and conditioned as described below. Bees remained harnessed in the tubes until the final US test (see below). Every afternoon, between 4:00 and 5:00 p.m., each bee was fed four times with 1 μl of 0.88 M sucrose solution.

### Behavioral Experiments

The conditioning experiments were conducted in front of an exhauster fan. The bees were removed from the storage box next to the exhauster fan thirty minutes prior to the conditioning and retention test. During conditioning an inter-trial interval (ITI) of 2 min was used. The CS consisted of a 5 s odor puff delivered by syringe. Odors were renewed daily by pipetting 4 μl of clove oil (Bombastus Werke AG, Freital) onto a filter paper (1 cm in diameter, MACHERY-NAGEL GmbH and Co. KG, Düren). The US consisted of a 1.25 M sugar solution delivered on a wooden toothpick. Conditioning trials started by placing the individual honeybee in front of the fan and were conducted as follows:

Forward conditioning trial: After allowing 10 s for placement in the experimental setup, the CS was presented for 5 s. Three seconds after the onset of the CS, the US was applied for 4 s. Eleven seconds after the offset of the US the bee was removed from the experimental setup.

Backward conditioning trial: After allowing 10 s for placement in the experimental setup the US was presented, which lasted for 4 s. Two seconds after the onset of the US, the CS was applied for 5 s. Eleven seconds after the offset of the CS the bee was removed from the experimental setup.

CS-only trial (memory retrieval): After allowing 10 s for placement in the experimental setup the CS was presented for 5 s. Thirteen seconds after the offset of the CS, the bee was removed from the experimental setup. A positive score was given if the bee’s proboscis crossed a virtual line between the open mandible tips during the CS presentation.

**Figure 1:**
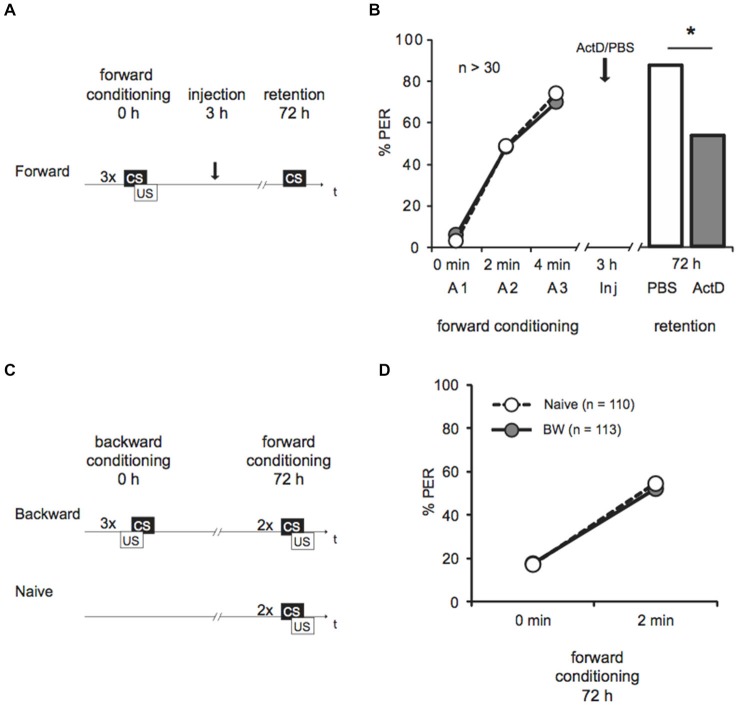
**Late long-term memory is formed after forward conditioning but not after backward conditioning. (A)** Two groups of honeybees were conditioned with three forward trials. Three hours after conditioning both groups were injected with either Act D or with the solvent PBS, 72 h after conditioning memory retention was tested. **(B)** The bees’ performance during the presentation of three forward conditioning trials does not differ between the two groups injected with either Act D (gray) or PBS (white) injection. The arrow indicates the time point of injection. Performance is lower in forward trained Act D-injected bees (gray) compared to PBS-injected bees (white) during the retention test at 72 h after conditioning. The asterisk indicates the significant differences (*p* < 0.05). **(C)** Retardation of acquisition assay. Honeybees were conditioned with three backward trials (backward) or were left untreated (Naive), 72 h after backward conditioning both groups received two forward conditioning trials. **(D)** The performance of bees during forward conditioning in the retardation of acquisition assay is not different between the Naive group (Naive, white) and the backward group (BW, gray).

After memory retrieval (Figures 1B, 4F), or the second forward conditioning phase of the retardation of acquisition assay (Figures 1D, 5B) the bees’ ability to extend the proboscis was tested by eliciting the proboscis extension response (PER) with the US (final US test). Only bees that responded with a PER to the US presentation where included in the data analysis.

#### Injection Protocol

One microliter Actinomycin D (Act D, 20 mM), Clasto-lactacystin β-lactone (β-lactone, 1 mM) or solvent were injected systemically into the honeybee’s flight muscle as shown in Felsenberg et al. (2011). Clasto-lactacystin β-lactone (β-lactone, Sigma-Aldrich, Munich) was dissolved in 10% (v/v) DMSO/PBS (PBS: 137 mM NaCl, 2.7 mM KCl, 10.1 mM Na2HPO4, 1.8 mM KH2PO4 at pH 7.2) to final a concentration of 1 mM. Actinomycin D (Act D, Sigma-Aldrich, Munich) was dissolved in PBS to a final concentration of 1.5 mM (Wüstenberg et al., 1998).

#### Amino Acid Sequence Alignment

We used the human monoubiquitin amino acid sequence (76 amino acids) derived from the three ubiquitin precursors: the UBC protein (AAH14880.1), the ubiquitin-40S ribosomal protein S27a precursor (NP_002945.1) or the ubiquitin-60S ribosomal protein L40 precursor (NP_003324.1) (Catic and Ploegh, 2005) as a query in the BLAST alignment tool (tblastn) on the *Apis mellifera* genome using the default settings.

#### Brain Dissection

The harnessed bees were anesthetized by cooling. The dissection was conducted on ice. The head capsule was opened and the glands and trachea were removed. The respective part of the brain was dissected and immediately frozen either in liquid nitrogen or on dry ice. The samples were stored at −80°C until usage.

#### Western Blot Analysis

**Figure 2:**
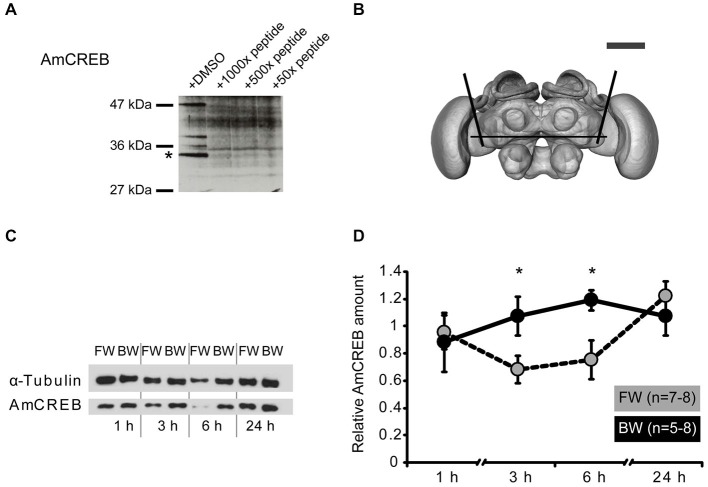
**The amount of AmCREB protein changes after conditioning. (A)** Peptide competition assay to test the specificity of the anti-human CREB antibody (see methods) used in this study. Before incubating a western blot of honeybee brain homogenate with an anti-human CREB antibody the antibody was preincubated with a 1000x, 100x, and 50x molar excess of a peptide from the C-terminus of AmCREB. This peptide is homologous to the antibody’s human epitope. Bands disappearing following the AmCREB peptide preincubation are regarded as AmCREB proteins. The 33 kDa band (marked with a star) is analyzed in the subsequent experiments. Left: Size of the prestained protein marker. **(B)** The central part of the bee brain was analyzed (picture taken from the honeybee standard atlas, http://www.neurobiologie.fu-berlin.de/beebrain/DownloadGeneral.html) (Brandt et al., 2005). Black lines delimit the dissected part. Scale bar ~300 μm (gray line). **(C,D)** Two groups of honeybees were conditioned with either three forward trials (FW, gray) or three backward trials (BW, black). The forward conditioned animals were selected according to a conditioned response (CR) in the third trial. The brains were dissected at 1 h, 3 h, 6 h and 24 h after conditioning and probed for their relative amount of AmCREB. **(C)** Representative western blot for the FW and BW at the respective time points. Following protein separation and blotting, the membrane was cut at approximately 50 kDa horizontally in two. The upper part (>50 kDa) was probed with the anti-α-tubulin antibody and the lower part (<50 kDa) with the anti-CREB antibody. **(D)** Quantification of the relative amount of AmCREB shows a decreased AmCREB amount 3 h and 6 h after conditioning in the FW group compared to the BW group. The asterisks indicate the significant differences (*p* < 0.05).

**Figure 3:**
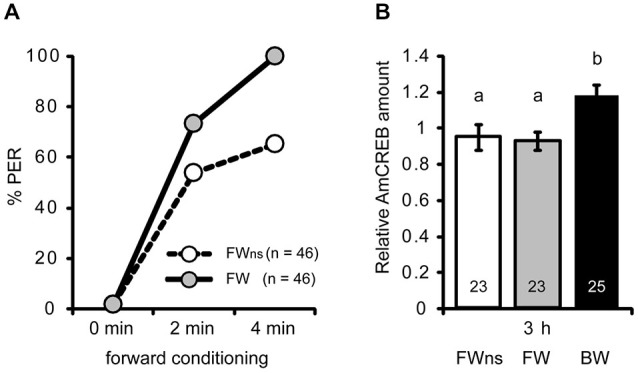
**The AmCREB amount depends on the timing of stimulus presentations during conditioning. (A)** Honeybees were forward conditioned with three trials. The forward conditioned animals were divided into two groups: one was selected according to a CR in the third trial (FW, gray) and the other remained unselected (FW_ns_, white). A third group received three backward trials (BW, trials not shown). **(B)** The brains were dissected 3 h after conditioning and analyzed for their relative AmCREB level. The quantification of the relative amount of AmCREB present shows a decreased amount in both forward conditioned groups compared to the backward conditioned bees. One sample consisted of two pooled brains. The numbers in the bars represent the sample size. The groups with unequal letters (a-b) differ significantly. The *P*-value was corrected due to multiple testing (*p* < 0.01). Whiskers represent the standard error.

**Figure 4:**
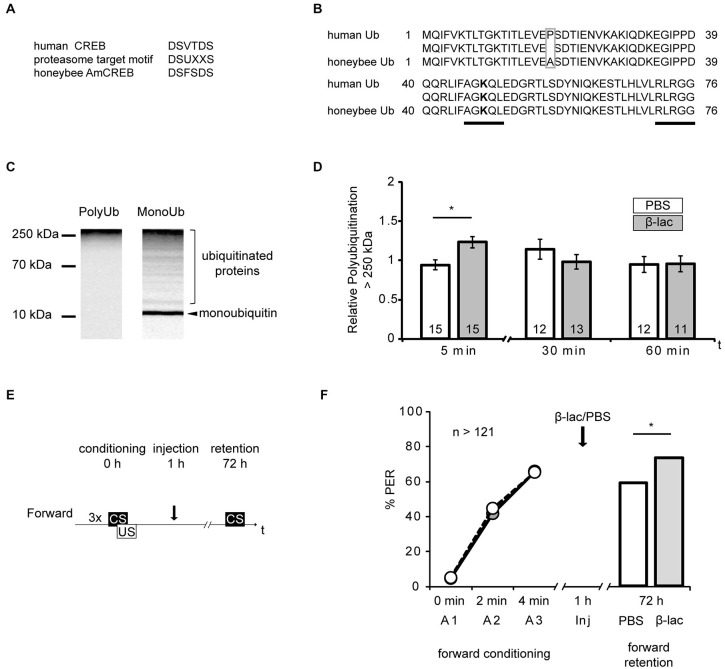
**Proteasome inhibition by β-lactone enhances late long-term memories (lLTM) upon forward conditioning. (A)** The proteasomal targeting motif of human CREB (Taylor et al., 2000) matches the amino acid sequence of AmCREB. **(B)** The comparison of the amino acid sequence of monoubiquitin derived from the human precursor UBC protein (Catic and Ploegh, 2005) compared to the predicted *Apis mellifera* ubiquitin revealed that these sequences are highly homologous. Only one out of 76 amino acids is different (gray box). The sequences that are critical for polyubiquitination (K48; bold, C-terminus) are identical (underlined in black). **(C)** Western blot analysis of mono- and poly-ubiquitin in bee brain lysate. A slot of a SDS-PAGE gel was loaded with bee brain homogenate. Following protein separation and blotting, the membrane was cut vertically from top to bottom in the middle of the lane and probed with antibodies detecting K48-linked poly-ubiquitin or mono-ubiquitin. The analysis shows that the monoubiquitin antibody detects a single band at ~10 kDa, whereas the antibody against K48-linked polyubiquitin does not detect this band. Both antibodies detect a smear. **(D–F)** Honeybees were injected with 1 mM of β-lactone (β-lac, gray) or the solvent PBS (white). The brains were dissected at 5 min, 30 min and 60 min after injection. **(D)** In order to examine the level of polyubiquitination, the membranes were cut into two on the level of the 70 kDa marker band. The upper part was probed with the antibody detecting K48-linked poly-ubiquitin and the lower part with the anti-α-tubulin antibody. Quantification shows that the signal is increased 5 min after injection in the β-lac groups compared to the PBS control group. **(E)** Honeybees were conditioned with three forward trials or three backward trials. One hour after conditioning, the honeybees were injected with either 1 mM of β-lactone (β-lac) or with the solvent PBS. Three days after conditioning, their memory was tested with one CS only trial. **(F)** The performance during the three forward conditioning trials did not differ in the bees subjected to the injection one hour later (arrow). Forward trained animals injected with β-lac (light gray) show increased performance during the memory test at 72 h after conditioning compared to the PBS control group (white). The asterisk indicates significant differences (*p* < 0.05).

Samples were defrosted, homogenized in 1x SDS-PAGE sample buffer (5x: 0.25 M Tris-Cl (pH 6.8), 50% (v/v) Glycerol, 5% (w/v) SDS, 0.05% (w/v) bromophenol blue, 0.25 M DTT) using a Teflon-glass homogenizer (experiments depicted in Figures 2, 3) or TSDG-ATP buffer (10 mM Tris-HCl, 25 mM KCl, 10 mM NaCl, 1 mM MgCl, 0.1 mM EDTA, 1 mM DTE, 2 mM ATP, 10% (v/v) glycerol, 0.1 mM NEM, 0.1 mM MG132, pH 7.5) using a Teflon-glass homogenizer or an automated homogenizer (Speed Mill Plus, Analytik Jena, Germany, 20 s min lysis tube P) (experiment shown in Figure 4). Homogenized samples were centrifuged for 15 min at 4°C at 14000 rpm.

Following homogenization in 1x SDS-PAGE sample buffer supernatants were heated to 95°C for 10 min and loaded onto the SDS-PAGE. After running the SDS-PAGE proteins were transferred to a nitrocellulose membrane (Optitran BA-S 83, Schleicher and Schuell, Dassel, Germany, Figures 2, 3).

Following homogenization in TSDG buffer and centrifugation, SDS-PAGE sample buffer was added to the supernatants of TSDG-ATP homogenates to a final concentration of 1x. Supernatants were heated to 95°C for 5 min, subjected to SDS-PAGE and transferred to a polyvinylidene difluoride membrane (Immun-Blot, Bio-Rad Laboratories, USA, experiments shown in Figure 4). Depending on the primary antibody used, membranes were blocked for 1 h at room temperature (RT) in different blocking solutions (for details see below). The primary antibody was diluted in the same blocking solution. The membrane was incubated overnight at 4°C with the primary antibody, washed three times for 10 min with TBST and incubated 1 h at RT with the secondary antibody diluted in blocking solution (see below). Subsequently, the membrane was washed three times with TBST and detected using the ECL system (PerkinElmer, Rodgau, Germany). Chemiluminescence signals were captured with a Kodak Biomax X-OMAT AR film (Figure 2) or LAS1000 camera and the software Image Reader LAS1000 2.60 (FUJIFILM Europe GmbH, Düsseldorf, Germany, Figures 3, 4). Band intensity was measured with MultiGauge version 3.0 (FUJIFILM Europe GmbH, Düsseldorf, Germany, Figure 3, 4) or ImageJ (Figure 2).

#### Primary Antibodies

**Anti-CREB antibody** (C21, #sc-186, rabbit polyclonal IgG, Santa Cruz Biotechnology, Heidelberg, Germany): Blocking was carried out with 3% BSA in Tris-buffered saline with Tween-20 (TBST: 10 mM Tris-HCl, 150 mM NaCl, 0.1% (v/v) Tween-20, pH 7.5) for 1 h at RT. The antibody was diluted 1: 2000 in blocking solution.

**Anti-K48-PolyUb antibody** (#05–1307, Anti-Ubiquitin Antibody, Lys48-Specific, clone Apu2, rabbit monoclonal, Merck Millipore, Merck KGaA, Darmstadt, Germany): Blocking was carried out in 5% nonfat milk powder solved in TBST for 1 h at RT. The antibody was diluted 1:1000 in blocking solution.

**Anti-monoubiquitin antibody** (#sc-8017, Ub Antibody, mouse monoclonal, clone P4D1, Santa Cruz Biotechnology, Dallas, USA): Blocking was carried out in 5% nonfat milk powder solved in TBST for 1 h at RT. The antibody was diluted 1:200 in blocking solution.

**Anti-α-Tubulin antibody** (#CP06, Anti-α-Tubulin Mouse monoclonal, clone DM1A, Calbiochem, Merck Millipore, Merck KGaA, Darmstadt, Germany): Blocking was carried out in 5% nonfat milk powder solved in TBST for 1 h at RT. The antibody was diluted 1:10000 in blocking solution.

#### Secondary Antibodies

**Anti-Mouse IgG-peroxidase conjugated** (#A3673, Sigma-Aldrich, St. Louis, USA): The antibody was diluted 1:10000 in 5% nonfat milk powder (Sucofin, TSI, Zeva, Germany) solved in TBST.

**Anti-Rabbit IgG-peroxidase conjugated** (#A6154, Sigma-Aldrich, St. Louis, USA): The antibody was diluted 1:10000 in 5% nonfat milk powder (Sucofin, TSI, Zeva, Germany) solved in TBST.

#### Peptide Competition Assay

In the peptide competition assay, the honeybee homolog of the human antigenic peptide, the AmCREB peptide AILENRNQTLIEELKSLKQLC (WITA GmbH, Teltow, Germany) was solved in DMSO. The anti-CREB antibody was incubated for 1 h at RT with 100×, 500× or 1000× molar excess of the AmCREB peptide in 500 μl 3% BSA solved in TBST. A membrane with honeybee homogenate was incubated overnight at 4°C with the pre-incubated antibody solution filled up with 3% BSA solved in TBST to the appropriate volume. AmCREB signals were detected as stated above.

#### Protein Quantification

**AmCREB** In order to examine the level of AmCREB, the membrane was cut at approximately 50 kDa horizontally in two. The upper part (>50 kDa) was probed with the anti-α-tubulin antibody and the lower part (<50 kDa) with the anti-CREB antibody as stated above.

**Polyubiquitin** In order to examine the level of polyubiquitination, the membranes were cut into pieces on the level of the 70 kDa marker band. The upper part was probed with the anti-K48-PolyUb antibody and the lower part with the anti-α-tubulin antibody.

For quantification, the values for individual α-tubulin samples from one blot were normalized to the mean of all tubulin samples from the same blot. The same was done for the AmCREB samples or polyubiquitin samples. The normalized value for the AmCREB sample (or polyubiquitin samples) was then divided by the corresponding normalized α-tubulin signal from the bees to control for differences in the amount of the loaded sample.

#### Statistics

For analysis of the behavioral data we used a G test for single comparisons of contingency tables (log-likelihood ratio for contingency tables) or Cochran’s Q test to analyze learning within a group over the acquisition trials. The Mann-Whitney *U* test (MWU) was used to analyze the differences in the quantification of the western blot results (Matsumoto et al., 2012). To test over multiple trials of behavior we used a repeated measurement ANOVA. All statistical tests were performed in Statistica (StatSoft, Hamburg, Germany) or Prism 6.0 (GraphPad Software, LaJolla, USA).

## Results

### Late LTM is Formed Following Forward Conditioning But Not Following Backward Conditioning

Our previous work on honeybees demonstrated that a memory for the excitatory and inhibitory properties of the CS can be retrieved 30 min and 24 h after backward conditioning with three trials presented with an ITI of 2 min (Felsenberg et al., 2013). Here we examined whether lLTM, characterized by the time point of its retrieval, 72 h following conditioning and its dependency on transcription, is formed following backward conditioning with the same backward conditioning protocol.

We compared lLTM formation following forward and backward conditioning. Thus in the first experiment we explored the formation of the lLTM following forward conditioning with three trials and an ITI of 2 min. Data from Lefer et al. (2012) suggest that injection of the transcriptional inhibitor Act D 3 h following forward conditioning with five forward conditioning trials shows the strongest effect on memory retention 72 h later (Lefer et al., 2012). Accordingly we choose this time point to inject the inhibitor to examine whether a transcription-dependent formation of lLTM following forward conditioning with three trials can be observed.

We forward conditioned bees with three trials and injected Act D or PBS 3 h later (Figure 1A). We demonstrated an increase of the bees’ CS response during conditioning from 3–6% to 70–74%, indicating learning (Figure 1B, Cochran’s Q test, PBS: *Q* = 37, d.f. = 2, *p* < 0.05, Act D: *Q* = 31, d.f. = 2, *p* < 0.05). In the retention test 72 h later, 89% bees of the control group responded to the CS. Bees injected with Act D showed a significantly lower response compared to the PBS-injected control group (Figure 1B, Act *D* = 55%, PBS = 89%, G test: *G* = 10.21, *p* < 0.05). This finding further confirms that this lLTM is dependent on transcriptional processes.

Next we asked whether lLTM for the excitatory or inhibitory properties acquired during backward conditioning is formed. It is not known yet, whether a 72 h memory can be retrieved following backward conditioning at all. Therefore we initially tested retention of a 72 h memory for the CS’ excitatory and the inhibitory properties following backward conditioning in untreated animals. We examined bees in a retardation of acquisition assay as previously described (Felsenberg et al., 2013). In this assay a naive group of bees and a backward conditioned group of bees are forward conditioned at the time point were memory retention shall be tested, which is 72 h after backward conditioning in our experiment. The naive bees’ response to the CS at the first acquisition trial reveals their spontaneous response to the CS, whereas the CS response of the backward conditioned group reveals memory retention for the CS’ excitatory properties generated by backward training. The bees’ response to the CS at the second trial allows determining the learning rate (with respect to the CS response at the first trial) and thus the fact that acquisition is generally slower after backward training. Thus, a retarded response to the second CS presentation compared to the naive group resembles memory retention for the CS inhibitory properties (Felsenberg et al., 2013).

We examined two groups of bees. One group was backward conditioned with three backward conditioning trials (BW), a second group remained untreated (Naive). Both groups were forward conditioned 72 h later (Figure 1C). The percentage of bees responding to the CS increased significantly from 18% at the first trial to 52% at the second trial (Figure 1D, rmANOVA: trials *F*_(1,221)_ = 107.12, *p* < 0.05) indicating successful learning. No significant difference between the backward conditioned and the naive groups was observed (Figure 1D, rmANOVA: treatment *F*_(1,221)_ = 0.01, *p* > 0.05). According to these results, long-term (72 h) retention was neither found for the CS excitatory properties nor for its inhibitory properties following three trial backward conditioning. We conclude that no 72 h memory is formed about either excitatory or inhibitory properties of the CS following backward conditioning. Thus, we did not further explore the susceptibility of memory formation for Act D 72 h following backward conditioning. Taken together these results demonstrate that memories formed following forward and backward conditioning with the same number of trials differ in their stability.

### Levels of a Honeybee CREB Homolog Differ Between Forward and Backward Conditioned Honeybees

It has been demonstrated that the strength of an associative memory depends on the transcription factor CREB and that the amount of the transcription factor CREB is crucial for memory stability (Yin et al., 1995; Josselyn et al., 2001; Tubon et al., 2013). Thus we next examined whether the amount of the transcription factor CREB in the honeybee brain differs between forward and backward conditioned animals. In order to detect AmCREB proteins we used an antibody raised against the C-terminus of human CREB.

This antibody detects several bands in honeybee brain homogenate including a band of approx. 33 kDa that disappeared after pre-incubation of the antibody with the homologous honeybee epitope (Figure 2A). The predicted size of the identified *Apis mellifera* CREB (AmCREB) variants lies between 26 and 33 kDa (Eisenhardt et al., 2006). Accordingly, the size of the 33 kDa band matches with the predicted size of an *Apis mellifera* CREB variant. Thus, we analyzed the 33 kDa band and from here on refer to it as *Apis mellifera* CREB (AmCREB).

We analyzed the AmCREB band at different time points after conditioning in the central upper part of the honeybee brain (Figure 2B). This part of the honeybee brain includes the mushroom bodies, a structure known to be involved in memory formation in insects (Erber et al., 1980; reviewed in Menzel, 2012). We compared the intensity of the AmCREB band 1 h, 3 h, 6 h, and 24 h following forward (FW) and backward (BW) conditioning. We conditioned bees with three trials with an ITI of 2 min. Only honeybees that showed a CR in the third acquisition trial were included in the FW group (“learners”). The central upper part of the brain was dissected 1 h, 3 h, 6 h and 24 h after conditioning.

We found that the relative intensity of the AmCREB band did not differ at 1 h after conditioning (Figures 2C,D, Mann-Whitney test (MWU): *U* = 15.00; *p* > 0.05) between forward and backward conditioned bees. However, compared to the BW group, the relative intensity was significantly decreased in the FW group at 3 h and 6 h after conditioning (MWU: *U*_3 h_ = 10.00; *p*_3 h_ < 0.05, *U*_6 h_ = 11.00; *p*_6 h_ < 0.05). One day after conditioning, the relative signal intensity of AmCREB in both groups was again at the same level (MWU test: *U* = 25; *p* > 0.05). Thus we conclude that the stimulus timing during conditioning impacts the amount of AmCREB in the honeybee brain.

### Differences in the Levels of AmCREB Depend on the Timing of Stimulus Presentations During Conditioning

Next we asked whether the observed difference in AmCREB levels between forward and backward conditioned honeybees is caused by the timing of stimulus presentations or whether it is correlated with the behavioral performance during learning. In the previous experiment (Figures 2C,D) animals were selected according to the CR at the last forward conditioning trial. According to Pamir et al. (2011) these animals can be regarded as bees that have already learned the association between CS and US (“learners”). In backward conditioning it is not possible to select “learners” according to their responsiveness to the CS at the last conditioning trial, because all bees respond to the preceding US presentation when the CS is presented. In the previous experiment we observed a significant difference in the AmCREB levels between forward and backward conditioned bees at 3 h following backward conditioning. Thus we repeated the above experiment for the 3 h time point but compared AmCREB level of a group of bees forward conditioned during three trials and selected for the occurrence of the CR at the third acquisition trial (FW), of an unselected group conditioned in a similar manner (FW_ns_), and of a group of bees backward conditioned during three trials (BW). Among the unselected bees, the percentage of bees showing a CR in the third trial was 65% (Figure 3A). The results of the western blot showed that in both forward conditioned groups, i.e., the selected (FW) and the unselected (FW_us_), the intensity of the AmCREB band is decreased compared to the backward conditioned group. The AmCREB band intensity in the two forward groups, the selected and the unselected, does not differ (Figure 3B, MWU test: *U*_BW vs. FW_ = 147.00; *p*_BW vs. FW_ < 0.01, *U*_BW vs. FWus_ = 155.00; *p*_BW vs. FWus_ < 0.01).

These results suggest that the amount of AmCREB present depends on the sequence of stimulus presentations but not on the learning rate during forward conditioning and therefore not on the learning success of an animal.

### AmCREB is a Potential Target for the Ubiquitin Proteasome System

Studies of vertebrate and invertebrate cells have suggested that CREB is degraded by the ubiquitin proteasome system (UPS) in response to physiological alterations, like hypoxia or altered levels of glucose (Taylor et al., 2000; Upadhya et al., 2004; Costes et al., 2009; Ozgen et al., 2010). During UPS-mediated proteolysis a target protein is tagged with a polyubiquitin tail via a multiple step enzyme cascade. The tagged protein is then degraded by the proteasome. The regulation of degradation of a specific substrate can be achieved by diverse mechanisms, e.g., allosteric changes due to peptide-protein interactions or phosphorylation at recognition sites (reviewed in Glickman and Ciechanover, 2002). The human CREB protein contains such a specific recognition site, a proteasomal targeting motif. Its phosphorylation is correlated with the polyubiquitin tagging of CREB and with its degradation by the UPS (Taylor et al., 2000). Accordingly, we investigated the possibility of CREB being a target of the UPS during memory formation. We examined whether the eight identified honeybee homologs of AmCREB (Eisenhardt et al., 2003, 2006) also contain the proteasomal target motif DSUXXS (where D is aspartic acid, S is serin, U is a hydrophobic amino acid and X is any amino acid) (Taylor et al., 2000). We analyzed these sequences with the BLAST alignment tool and identified in all eight AmCREB variants the motif DSFSDS (Figure 4A, position of motif: for AmCREB 1, AmCREB 4, and AmCREB 5 at positions 121–126; for AmCREB 2, AmCREB 3, AmCREB 7 and AmCREB 8 at positions 89–94; for AmCREB 6 at position 83–88). Thus we hypothesize that AmCREB is a potential target protein of the proteasome pathway.

### Proteasome Inhibition Strengthens lLTM for the CS Excitatory Properties Following Forward Conditioning

Above we hypothesize that AmCREB is a target protein of the UPS in the honeybee brain. If this hypothesis holds true and AmCREB is degraded by the proteasome within 1–3 h following forward but not backward conditioning, blocking of the proteasome during this time period should impact lLTM formation after forward but not backward conditioning.

Next we tested the hypothesis. We previously demonstrated that both inhibitors of the proteasome, β-lactone and MG132, block the proteasome activity in honeybee brain lysate in a dose-dependent manner (Felsenberg et al., 2014). Here we examined the time course of proteasome inhibition following the injection of 1 mM β-lactone, a dose that blocks >80% of the chymotrypsin-like proteasome activity (Felsenberg et al., 2014). We examined the level of polyubiquitinylated proteins present in the brain following β-lactone injection assuming that an enrichment of polyubiquitinated proteins indicates an inhibition of the proteasome.

Specific polyubiquitin chains linked via the K 48 and G 76 tag proteins for degradation by the UPS (Glickman and Ciechanover, 2002). By using the BLAST alignment tool, we showed that human ubiquitin is highly homologous to the predicted protein sequence of *Apis mellifera* ubiquitin (Figure 4B). In fact only one amino acid is different (alanine instead of proline at position 19) between the human monoubiquitin and the predicted honeybee ubiquitin sequence. Accordingly, the amino acid sequence flanking K 48 is conserved in the honeybee ubiquitin. We therefore analyzed monoubiquitin and polyubiquitins linked via K48 in western blot analysis using human ubiquitin antibodies (Figure 4C). The monoubiquitin antibody detected a signal with several bands and a smear distributed over the lane, with a prominent band observed around 10 kDa. We assumed that the smear and higher bands represented ubiquitinated and polyubiquitinated proteins, and that the band at 10 kDa represented the honeybee monoubiquitin. Moreover, the antibody against K48-linked polyubiquitin detects signals at a higher mass range, but not at the 10 kDa band.

These results imply that ubiquitin exists in the honeybee brain and that the antibody against the K48-linked polyubiquitin can be used to detect polyubiquitinated honeybee proteins.

In order to control for the effect of the systemically injected β-lactone, we injected bees with either 1 μl of 1 mM β-lactone or the solvent (PBS). We dissected the central brain, i.e., the brain devoid of the optical lobes, at 5 min, 30 min and 60 min after injection and analyzed the homogenates in a western blot analysis to reveal the relative amount of K48-linked polyubiquitin. We detected the strongest signal of polyubiquitin above the 250 kDa marker band. Therefore, we used this region for the analysis. The polyubiquitin signal >250 kDa was significantly higher in brains of animals injected with β-lactone than in brains of the control group at 5 min after injection (Figure 4D, Mann-Whitney U test: *U* = 48.00, *p* < 0.05). No significant differences were observed at 30 min or 60 min after injection (Mann-Whitney U test: 30 min; *U* = 65.00, *p* > 0.05, 60 min; *U* = 69.00, *p* > 0.05). Accordingly, we observed an enrichment of polyubiquitin in the honeybee brain shortly after systemic injection of β-lactone.

Next we analyzed the effect of proteasome inhibition 1 h after forward conditioning on lLTM formation. One hour after conditioning, honeybees received an injection with β-lactone or the solvent. Three days later, memory retention was tested (Figure 4E). In the forward conditioned group the percentage of CR was significantly higher in β-lactone-injected bees compared to PBS-injected ones (Figure 4F, β-lactone = 74%, PBS = 59%, G test: *G* = 6.24, *p* < 0.05).

Taken together these results demonstrate that blocking proteasome activity with 1 mM β-lactone 1 h after forward conditioning increases lLTM formation about the CS excitatory properties.

### The Proteasome Does Not Play a Role in Gating lLTM Formation Following Backward Conditioning

**Figure 5:**
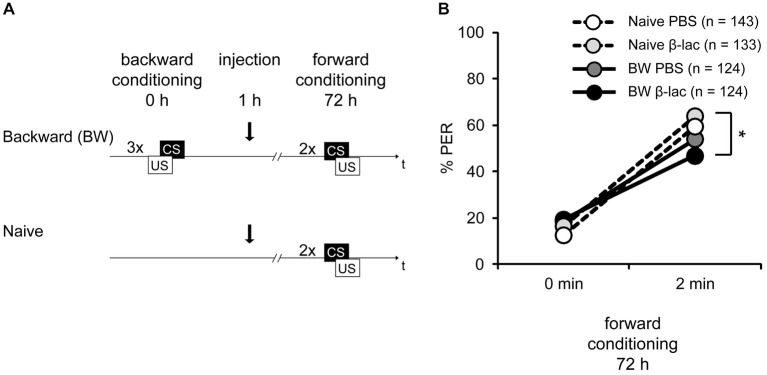
**Proteasome activity does not gate lLTM for the CS excitatory and inhibitory properties following backward conditioning. (A)** Honeybees were conditioned with three backward trials (BW) or remained naive (Naive). Three hours after conditioning, both groups were injected with either 1 mM of β-lactone (β-lac) or with the solvent PBS. Three days after conditioning all groups received two forward trials. **(B)** The performance of bees during the retardation test shows that backward conditioned bees injected with PBS (Naive PBS, white and BW PBS, dark gray) were not different from each other and from the respective β-lac groups, indicating that no memory for the CS inhibitory properties was formed. However, β-lac-injected bees (BW β-lac, black) show a reduced response in the second acquisition trial compared to the Naive β-lac-injected bees (light gray) suggesting that proteasome activity to a small extent impacts the acquisition of excitatory AND inhibitory properties. The asterisk indicates significant differences of the* post hoc* test (*p* < 0.05).

Above we demonstrated that β-lactone increases the formation of a lLTM about the excitatory properties of the CS. This suggests that the proteasome plays a role in gating lLTM formation. Thus we next asked whether this could be observed following backward conditioning. We backward conditioned bees with three trials (BW) while another group of bees remained naive (Naive). One hour after conditioning both groups were divided into two groups each receiving either β-lactone or PBS injections. Three days later all four groups were forward conditioned with two conditioning trials (Figure 5A).

During forward conditioning a significant increase in the bees’ responsiveness was observed between the first and the second trial (Figure 5B, rmANOVA: trial *F*_(1,525)_ = 302.40, *p* < 0.05) indicating successful learning. No significant difference in CS responsiveness between the backward conditioned and the naive groups (rmANOVA: training *F*_(1,522)_ = 1.63, *p* > 0.05), and the β-lactone- and the PBS-injected bees was found (rmANOVA: injection *F*_(1,525)_ = 0.01, *p* > 0.05). However a significant difference between the backward conditioned bees and the naive bees can be observed at the second conditioning trial (rmANOVA: trial × training *F*_(1,525)_ = 12.35, *p* < 0.05). The CS responsiveness differs significantly at the second forward trial between the β-lactone-treated BW and the β-lactone-treated naive group (Figure 5B; Tukey HSD *post hoc* test_(β-lactone naive 2nd trial vs. β-lactoneBW 2nd trial),_ *p* < 0.05), but not between the PBS-treated BW conditioned and naive group (Figure 5B; Tukey HSD *post hoc* test_(PBS BW 2nd trial vs. PBS naive 2nd trial)_ *p* > 0.05). At the second forward conditioning trial no significant difference in CS responsiveness between the backward conditioned animals injected with β-lactone and PBS was observed (Tukey HSD *post hoc* test_(β-lactone BW 2nd trial vs. PBS BW 2nd trial)_ *p* > 0.05) and no significant difference was observed between the naive animals injected with β-lactone or PBS (Tukey HSD *post hoc* test_(β-lactone naive 2nd trial vs. PBS naive 2nd trial)_ *p* > 0.05). Taken together, these results show no retardation of acquisition in PBS-treated bees. Moreover, no significant difference was observed between β-lactone-injected and PBS-injected animals that were either backward conditioned or remained naive. Thus, as shown before (Figure 1D), no lLTM was formed about the inhibitory properties of the CS. Moreover, we conclude that β-lactone does not gate the formation of lLTM for the CS inhibitory properties. However, we do observe a significant difference in the retardation of acquisition assay when we analyze both β-lactone-injected groups, naive animals and backward conditioned animals. We conclude that two small effects of β-lactone add up: an enhancement of the CS responsiveness of naive animals during forward conditioning and an enhancement of memory strength for the CS inhibitory properties acquired during backward conditioning. But despite the rather big sample size the effect appears to be small and is not significantly different to all the parallel controls.

## Discussion

### Memories Formed Upon Forward Conditioning and Backward Conditioning Differ in their Stability

In line with several previous studies in the honeybee we demonstrate a transcription-dependent lLTM for the excitatory properties of the CS 72 h following forward conditioning (Wüstenberg et al., 1998; Friedrich et al., 2004; Hourcade et al., 2010; Felsenberg et al., 2011; Lefer et al., 2012). In contrast, after backward conditioning we observed no lLTM retention of the CS’ excitatory or inhibitory properties.

Previous work in honeybees showed that backward conditioning induces a memory for the inhibitory CS properties that can be retrieved 30 min and 24 h later (Hellstern et al., 1998; Felsenberg et al., 2013). In addition, a parallel memory for the excitatory CS properties can be retrieved 24 h, but not 30 min after backward conditioning (Felsenberg et al., 2013). Thus, in principle, two memories—a memory about the excitatory CS properties and a memory about the inhibitory CS properties—are formed following backward conditioning. The fact, that we cannot retrieve any memory 72 h after backward conditioning suggests that no lLTM is formed following appetitive backward conditioning. Thus memories formed after backward conditioning with three trials might be less stable than memories formed after forward conditioning with the same trial number.

This result resembles findings from aversive conditioning in the fruit fly *D. melanogaster* (Diegelmann et al., 2013). In *D. melanogaster* forward conditioning with an aversive US, termed “punishment learning” results in avoidance of the conditioned odor (Quinn et al., 1974; Tully and Quinn, 1985). In contrast, after backward conditioning with an aversive US flies approach the conditioned odor (Tanimoto et al., 2004). This backward learning situation is termed “relief learning” (Tanimoto et al., 2004; Yarali et al., 2008). A memory following aversive forward conditioning of flies can be retrieved up to 24 h later, whereas memories formed upon relief learning can only be retrieved up to 75 min after backward conditioning (Diegelmann et al., 2013). Since these findings are in line with our results, we conclude that, irrespective of the valence of the unconditioned stimulus, memories formed upon backward conditioning are less stable than memories formed following forward conditioning.

### Stimulus-Timing Impacts AmCREB Levels

Our data shows for the first time that the level of CREB protein is differentially altered following forward compared to backward conditioning. So, what might be the cause for these observed differences in the amount of AmCREB? Two explanations appear plausible. First, forward and backward conditioning might lead to memories of different strength and might therefore result in different levels of AmCREB, irrespective of whether the CS acquired inhibitory or excitatory properties. However, we show that the amount of AmCREB present is not different in two forward trained groups that were selected according to the percentage of animals responding with a CR at the last forward conditioning trial (60% and 100%). The percentage of animals responding to the conditioned odor with a CR is thought to reflect the associative strength acquired by the CS during conditioning (Rescorla and Wagner, 1972). Thus, we conclude that the CS associative strength does not impact the AmCREB level. However, both forward conditioned groups exhibit lower levels of AmCREB compared to the backward conditioned bees. This result indicates that the differences in AmCREB level following forward and backward conditioning might be a consequence of the different timing of stimulus presentations between the two conditioning protocols.

How might the stimulus sequence impact the underlying molecular mechanisms? One possible mechanism has been proposed for the fruit fly *D. melanogaster*. According to a computational study, forward conditioning enhances the activity of the type I adenylyl cyclase AC I, which has been shown to act as a coincidence detector integrating CS- and US-dependent molecular processes in the fruit fly (Tomchik and Davis, 2009; Gervasi et al., 2010; Boto et al., 2014). Backward conditioning, in contrast, leads to reduced AC I activity and thus a cAMP level below the cAMP level of control animals (Yarali et al., 2012). Thus, forward conditioning but not backward conditioning might induce cAMP-dependent processes that result in the activation of CREB (Kandel, 2001) following forward but not backward conditioning. But what might be the reason for a decrease of AmCREB 3 h 6 h following forward conditioning?

### A Learning-Induced Decrease of CREB by the UPS Following Forward Conditioning?

Inhibition of the proteasome 1 h following forward conditioning enhances lLTM formation for the CS excitatory properties. This finding is in line with previous studies showing an enhancement of LTM formation following proteasome inhibition in honeybees and vertebrates (Yeh et al., 2006; Felsenberg et al., 2012, 2014), indicating that the UPS negatively regulates LTM strength following learning.

Interestingly, increasing the amount of CREB enhances the formation of LTM (Han et al., 2008; Sekeres et al., 2012; Tubon et al., 2013) suggesting that the CREB amount is correlated with memory strength. Accordingly, we hypothesize that the role of the UPS in regulating memories strength and the decrease of CREB following forward conditioning might be interrelated.

Based on our results we developed the following model: CREB is activated following forward but not backward conditioning possibly by the cAMP-dependent signaling cascade (see above and Yarali et al., 2012) such that transcription of CREB-dependent genes takes place only following forward conditioning. At the same time CREB is modified, e.g., by phosphorylation (Taylor et al., 2000), to be subsequently degraded by the UPS. CREB degradation terminates CREB-dependent transcription thereby regulating the LTM’s strength following forward conditioning. In support of this model is the finding that enhancing CREB activity leads to an enhancement of LTM strength following forward conditioning (Restivo et al., 2009; Suzuki et al., 2011; Vetere et al., 2011). However, termination of CREB-dependent transcription does not mean that transcription of memory genes depending on other transcription factors (Alberini, 2009) is stopped. This explains why inhibition of transcription with Act D 3 h after conditioning inhibits lLTM formation although we propose a termination of CREB-dependent transcription 3 h after forward conditioning. Moreover, several proteins have been identified that are degraded by the UPS following learning (Lee et al., 2008; Jarome et al., 2011). Thus in addition to CREB, the UPS might target other proteins involved in lLTM formation following forward conditioning.

In our model the amount of CREB remains unaltered following backward conditioning, because it is not modified in response to conditioning to be subsequently degraded by the UPS. However, we cannot exclude that other proteins are degraded by the UPS in response to backward conditioning. In fact, above we demonstrate that treating naive and backward conditioned bees with the proteasome inhibitor β-lactone 1 h after backward conditioning results in a small but significant retardation of acquisition in both inhibitor groups, but not the control groups. We concluded that two small effects of β-lactone add up: first an enhancement of the CS responsiveness of naive bees and second a decrease of the CS responsiveness of backward conditioned bees during forward conditioning. This finding might point towards an involvement of the UPS pathway in restriction of memory formation upon backward conditioning, although it remains unclear why in this case also the responsiveness of naïve animals should be enhanced during forward conditioning. Moreover, despite the rather big sample size the effect appears to be small and is not significantly different to all the parallel controls.

### Learning-Induced AmCREB Alterations

Three hours and six hours after forward conditioning we observe a reduced level of the honeybee’s CREB homolog, AmCREB, compared to backward conditioning. Our finding of an alteration of the AmCREB level following conditioning, which depends on the timing of CS and US, is supported by several studies in invertebrate and vertebrate animals. In the basolateral nucleus of the rat amygdala (BLA) forward conditioning in a conditioned taste aversion paradigm, results in the expression of Arc/Arg 3.1, a target gene of CREB (Barot et al., 2008; Kawashima et al., 2009). Moreover, a high number of BLA cells showed Arg/Arg 3.1 expression as a result of convergent CS and US activation during forward conditioning, whereas the number of cells detected following backward conditioning was not significantly different from the CS-only and US-only controls. Moreover, in fear conditioning induction of Arg/Arg3.1 expression is enhanced in the lateral amygdala in comparison to backward conditioning and naive animals (Chau et al., 2013).

A similar result was obtained in the fruit fly, *Drosophila melanogaster*, where in subsets of ellipsoid body neurons and mushroom body intrinsic cells CREB-dependent transcriptional activity was enhanced following forward compared with backward conditioning. However, in a different subset of these neurons the opposite was observed, namely a decrease of CREB-dependent transcription within the first couple of hours after forward conditioning compared to backward conditioning, which returned to baseline 24 h later (Zhang et al., 2015). This time course of CREB-dependent transcription parallels the time course of the decrease of CREB amount observed in our study. It remains to be demonstrated whether the alteration of CREB-dependent transcription observed by Zhang et al. (2015) is due to an alteration of CREB phosphorylation or CREB amount.

Changes in the amount of CREB, as demonstrated in our study, might act as an additional level of control of CREB-dependent gene transcription following learning. Support for this hypothesis comes from studies in the sea hare *Aplysia californica* where the induction of long-term facilitation (LTF), the synaptic correlate of non-associative long-term memory, leads to an alteration of the CREB level (Liu et al., 2008, 2011a,b). Both a CREB activator and a CREB repressor show a biphasic alteration following LTF induction with an initial increase of the protein amount followed by a decrease below the level of untreated control animals and a second increase of the proteins 18 h following LTF induction (Liu et al., 2008, 2011a,b). Interestingly, these dynamics of CREB have been suggested to be regulated via positive and negative feedback loops (Song et al., 2007) and the UPS has been shown to degrade the *Aplysia* CREB repressor suggesting a role of the UPS in these feedback loops (Upadhya et al., 2004).

In summary, we demonstrated that the stability of LTM formed after forward and backward conditioning with the same number of conditioning trials is different: lLTM retention can be observed only after forward conditioning but not after backward conditioning. Accordingly we observed that transcription and proteasome activity play a role in memory formation following forward conditioning, but not following backward conditioning. Moreover we found a difference in the dynamics of the AmCREB level following forward and backward conditioning. Our results provide evidence that the AmCREB level is regulated by the proteasome. A proteasome-dependent mechanism might regulate lLTM strength following forward conditioning—a process that is not required in more rapidly decaying memories formed after backward conditioning.

## Conflict of Interest Statement

The authors declare that the research was conducted in the absence of any commercial or financial relationships that could be construed as a potential conflict of interest.
